# Evaluation of bone changes in medication-related osteonecrosis of the jaw by using fractal analysis on CBCT and panoramic radiographs

**DOI:** 10.1186/s12903-025-07503-z

**Published:** 2025-12-09

**Authors:** Mehmet Egemen Aydemir, Derya Yıldırım, Özlem Görmez, Hikmet Orhan

**Affiliations:** 1 Department of Dentomaxillofacial Radiology, Faculty of Dentistry, Burdur Mehmet Akif Ersoy University, Burdur, 15100 Turkey; 2 Department of Dentomaxillofacial Radiology, Faculty of Dentistry, Süleyman Demirel University, Isparta, 32200 Turkey; 3 Department of Basic Medical Sciences, Division of Biostatistics and Medical Informatics, Faculty of Medicine, Süleyman Demirel University, Isparta, 32200 Turkey

**Keywords:** Medication-related osteonecrosis of the jaw (MRONJ), Fractal analysis, Cone beam computed tomography (CBCT), Panoramic radiography (OPG), Bone microarchitecture, Early detection, Maxillofacial radiology

## Abstract

**Background:**

Medication-related osteonecrosis of the jaw (MRONJ) can present with subtle trabecular alterations before overt radiographic or clinical signs become evident. This study evaluated the ability of fractal analysis on cone-beam computed tomography (CBCT) and panoramic radiographs (OPGs) to detect such early changes, and compared the diagnostic performance of the two imaging modalities.

**Methods:**

This retrospective analysis included MRONJ patients with a history of intravenous zoledronic acid therapy and age- and sex-matched healthy controls, all of whom had both CBCT and OPG examinations. Fractal dimension (FD) values were calculated from 8 standardized regions of interest (ROIs) on CBCT and 6 on OPG images using ImageJ^®^ software. Group differences were assessed, and correlations with sex and medication duration were analyzed.

**Results:**

A total of 54 patients (26 MRONJ, 28 controls) met the inclusion criteria and were included in the analysis. CBCT revealed significantly lower FD values in MRONJ patients compared to healthy controls, with reductions ranging from 0.02 to 0.05 in various ROIs (*p* < 0.05), indicating trabecular bone alterations. OPG images, in contrast, failed to demonstrate statistically significant group differences. A significant negative correlation between FD values and medication duration was found in the mandibular corpus, tuberosity, and mental foramen regions on CBCT, but not on OPG. Sex-related differences were detected in certain ROIs, but were inconsistent across sites.

**Conclusions:**

CBCT-based fractal analysis demonstrated markedly greater sensitivity than OPG in detecting both localized and widespread trabecular changes in MRONJ, including alterations invisible to conventional imaging. These results underscore CBCT fractal analysis as a promising noninvasive tool for early detection and monitoring of MRONJ-related bone changes.

## Background

Bone homeostasis depends on a balance between formation by osteoblasts and resorption by osteoclasts. Antiresorptive and antiangiogenic agents, such as bisphosphonates, inhibit osteoclast activity and are effective in treating osteoporosis and bone metastases, but may cause medication-related osteonecrosis of the jaw (MRONJ) [1–3]. This chronic condition, characterized by necrotic bone exposure in the oral cavity, negatively affects quality of life and presents considerable clinical challenges [4, 5].

Radiographic signs of MRONJ include osteosclerosis, osteolysis, thickened lamina dura, subperiosteal bone deposition, and delayed socket healing [6]. Panoramic radiography (OPG) is commonly used due to its accessibility, but its two-dimensional nature limits assessment of trabecular architecture. Cone-beam computed tomography (CBCT) offers high-resolution, three-dimensional imaging with superior visualization of bone microstructure. However, comparative studies using both modalities in the same patient cohort are scarce, especially those evaluating bone that appears radiographically healthy [7, 8].

Fractal analysis (FA) quantitatively measures trabecular complexity, with FD values reflecting the degree of structural irregularity. While higher FD values are generally associated with more complex trabecular patterns, pathological conditions such as MRONJ may present with reduced FD values due to loss of trabecular organization despite areas of apparent sclerosis [9–11]. Beyond these numerical metrics, FA enables the detection of subtle microarchitectural changes not appreciable on visual inspection alone, potentially allowing earlier diagnosis of disease-related bone alterations [12]. This quantitative sensitivity is a major strength, allowing FA to serve as a noninvasive tool to capture subclinical deterioration that precedes overt radiographic or clinical signs [13].

FA has been applied in various medical fields, including dentistry, to evaluate trabecular patterns in osteoporosis, peri-implantitis, and other bone-related conditions [14, 15]. In MRONJ research, most FA studies have focused on necrotic areas or used a single imaging modality [7, 8]. However, the literature presents conflicting findings: while some CBCT-based studies report increased FD values, interpreted as reparative or sclerotic changes, others consistently demonstrate reduced FD values, suggesting trabecular loss. Such inconsistencies underscore key limitations of FA, namely its high sensitivity to imaging parameters, ROI selection, and analysis protocols. These methodological dependencies complicate cross-study comparisons and highlight the need for standardized, robust protocols and comparative designs [8, 12, 16].

Histologic evidence suggests that antiresorptive therapy can induce microarchitectural changes even in clinically unaffected bone, likely due to its systemic skeletal effects [17]. Therefore, assessing both necrotic and radiographically healthy-appearing regions using multiple anatomical sites and imaging modalities (CBCT and OPG) is crucial to clarify the true extent of bone deterioration. Given the severe morbidity associated with MRONJ, establishing reliable imaging methods for early detection of trabecular alterations has direct clinical relevance, as it may guide timely interventions and improve patient outcomes.

Previous research has compared fractal parameters across imaging modalities. Saberi et al. reported that while metallic objects did not significantly influence FD values on CBCT, FD values differed significantly among CBCT, periapical, and panoramic radiographs, with periapical radiographs showing the highest diagnostic reliability [18]. Similarly, Magat and Sener found that CBCT and panoramic radiographs yielded significantly different FD and BAF values, concluding that panoramic radiographs provided superior image resolution for fractal analysis compared with CBCT [19]. These studies highlight that FD can be derived from both CBCT and OPG, but also demonstrate that the modalities are not interchangeable, thereby supporting the relevance of our direct comparison in MRONJ patients.

This study aimed to compare the diagnostic performance of CBCT and OPG in detecting trabecular bone changes by analyzing fractal dimension (FD) values in MRONJ patients and matched healthy controls across multiple regions of interest (ROIs). We hypothesized that CBCT-derived FD values, owing to its three-dimensional capability, would reveal significant trabecular loss in MRONJ patients, whereas OPG would not demonstrate such differences. The null hypothesis was that FD values would not differ significantly between groups, irrespective of bone region or imaging modality.

## Methods

This retrospective study was approved by the Süleyman Demirel University Clinical Research Ethics Committee (Approval No: 72867572-050.01.01.04–40137, dated March 26, 2021) and conducted in accordance with the Declaration of Helsinki.

This study included 26 patients with MRONJ who had received intravenous zoledronic acid therapy for metastatic bone cancer, and 28 age- and sex-matched healthy controls. MRONJ diagnoses and staging were established clinically and radiographically as stage 2 or stage 3 according to the American Association of Oral and Maxillofacial Surgeons criteria [20]. All MRONJ patients were examined at the Department of Dentomaxillofacial Radiology, Faculty of Dentistry, Süleyman Demirel University. Radiographic data were obtained from the department’s radiological database, and clinical examination findings were retrieved from the patients’ records maintained in the department. Both OPG (Planmeca Promax, Helsinki, Finland) and CBCT (Planmeca Promax 3D Mid, Helsinki, Finland) images were obtained as part of the diagnostic workup.

Inclusion criteria were: (1) availability of both CBCT and OPG images, (2) age ≥ 18 years, and (3) absence of systemic diseases affecting bone metabolism, such as osteoporosis, hyperparathyroidism, or Paget’s disease, as confirmed by review of medical records. These criteria ensured that any differences in FD values could be attributed to MRONJ rather than underlying metabolic disorders. Exclusion criteria included: (1) an interval of more than three months between OPG and CBCT acquisition to ensure imaging consistency, (2) presence of CBCT artifacts compromising trabecular bone evaluation, and (3) systemic diseases or extensive jaw lesions (e.g., large cysts or tumors) that could affect jawbone integrity.

Based on an estimated effect size of 0.72 from previous FA studies in bone conditions [7, 8], a minimum of 25 participants per group was required to achieve 80% statistical power at a 5% significance level (α = 0.05). The calculation was performed using G*Power software (version 3.1, Heinrich Heine University, Düsseldorf, Germany). Initially, both the MRONJ and control groups included 28 participants; however, two MRONJ cases were excluded due to missing data, resulting in 26 MRONJ patients and 28 controls in the final analysis.

All OPG and CBCT scans were obtained using standardized imaging protocols to ensure consistency. Both modalities were performed on the same imaging unit. Panoramic radiographs were acquired at 66 kVp, 9 mA, with an exposure time of 16 s. CBCT scans were obtained at 90 kVp, 10 mA, with an exposure time of 14 s, a voxel size of 400 μm, and a field of view (FOV) of 20 × 10 cm. All images were processed using Planmeca Romexis software (version 3.8.3; Helsinki, Finland) and exported in 16-bit TIFF format for subsequent analysis.

ROIs were selected from both necrotic bone areas and radiographically healthy-appearing bone in anatomically distinct regions of the mandible and maxilla. Necrotic bone ROIs were identified based on previously documented clinical diagnoses in the patient records (areas of exposed bone or intraoral/extraoral fistulas), corroborated by radiographic features such as osteosclerosis, osteolysis, or cortical disruption. For radiographically healthy-appearing ROIs, the selected regions were reviewed by two oral and maxillofacial radiologists (MEA and DY), and consensus was reached to confirm the absence of MRONJ-related radiographic changes. This approach minimized the risk of selection bias and ensured the accuracy of ROI classification.

In the MRONJ group, if a predefined standardized ROI for radiographically healthy-appearing bone overlapped with a necrotic or sclerotic lesion, the ROI was shifted to the nearest anatomically comparable region. The adjusted sites were carefully reviewed by two oral and maxillofacial radiologists (MEA and DY) in consensus to confirm the absence of MRONJ-related radiographic features, thereby maintaining both methodological consistency and data integrity.

A total of 14 standardized ROIs were analyzed: 8 from CBCT (7 radiographically healthy-appearing bone regions and 1 necrotic region) and 6 from OPG (5 radiographically healthy-appearing bone regions and 1 necrotic region).

**Fig. 1:**
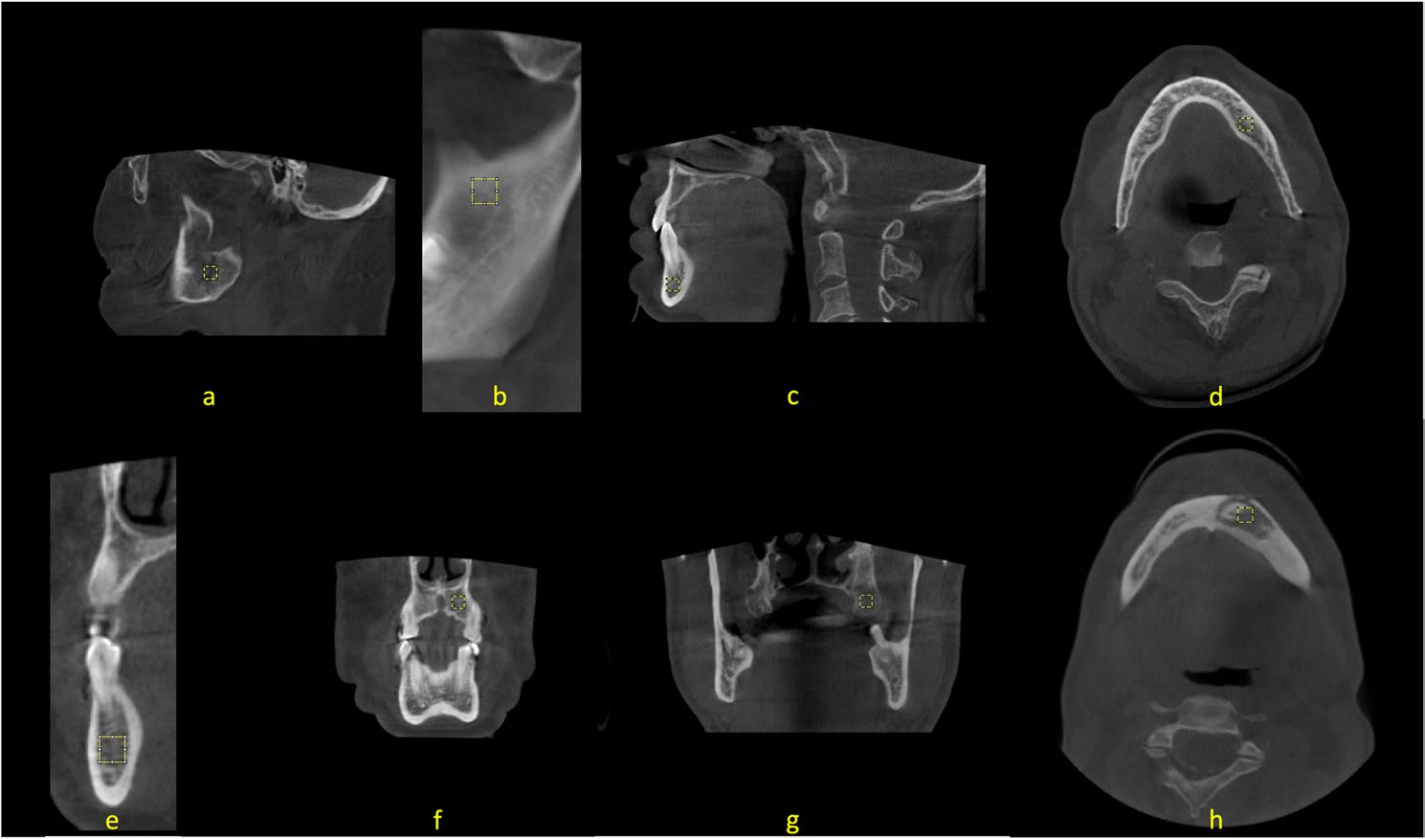
Regions of interest (ROIs) identified on CBCT images. **a. **ROI 1: Mandibular angle region – parasagittal view (30×30 px). **b. **ROI 2: Mandibular foramen superior region – panoramic curved planar reformatted (CPR) view (30×30 px). **c.** ROI 3: Mental foramen anterior region – parasagittal view (30×30 px). **d.**ROI 4: Superior to the inferior alveolar canal – axial view (30×30 px). **e.**ROI 5: Mental foramen medial region – cross-sectional CPR-perpendicular view (30×30 px). **f**. ROI 6**:** Maxillary canine periapex – coronal view (30×30 px). **g**. ROI 7: Maxillary tuberosity – coronal view (30×30 px). **h**. ROI 8: Necrotic bone area – axial view (30×30 px, MRONJ group only)

For CBCT images (Fig. 1; all ROIs = 30 × 30 pixels):

- ROI 1 – Mandibular angle (parasagittal view).
- ROI 2 – Mandibular foramen superior region (panoramic CPR view).
- ROI 3 – Mental foramen anterior region (parasagittal view).
- ROI 4 – Superior to the inferior alveolar canal (axial view).
- ROI 5 – Mental foramen medial region (cross-sectional CPR-perpendicular view).
- ROI 6 – Maxillary canine periapex (coronal view).
- ROI 7 – Maxillary tuberosity (coronal view).
- ROI 8 – Necrotic bone area (axial view, MRONJ group only).

**Fig. 2:**
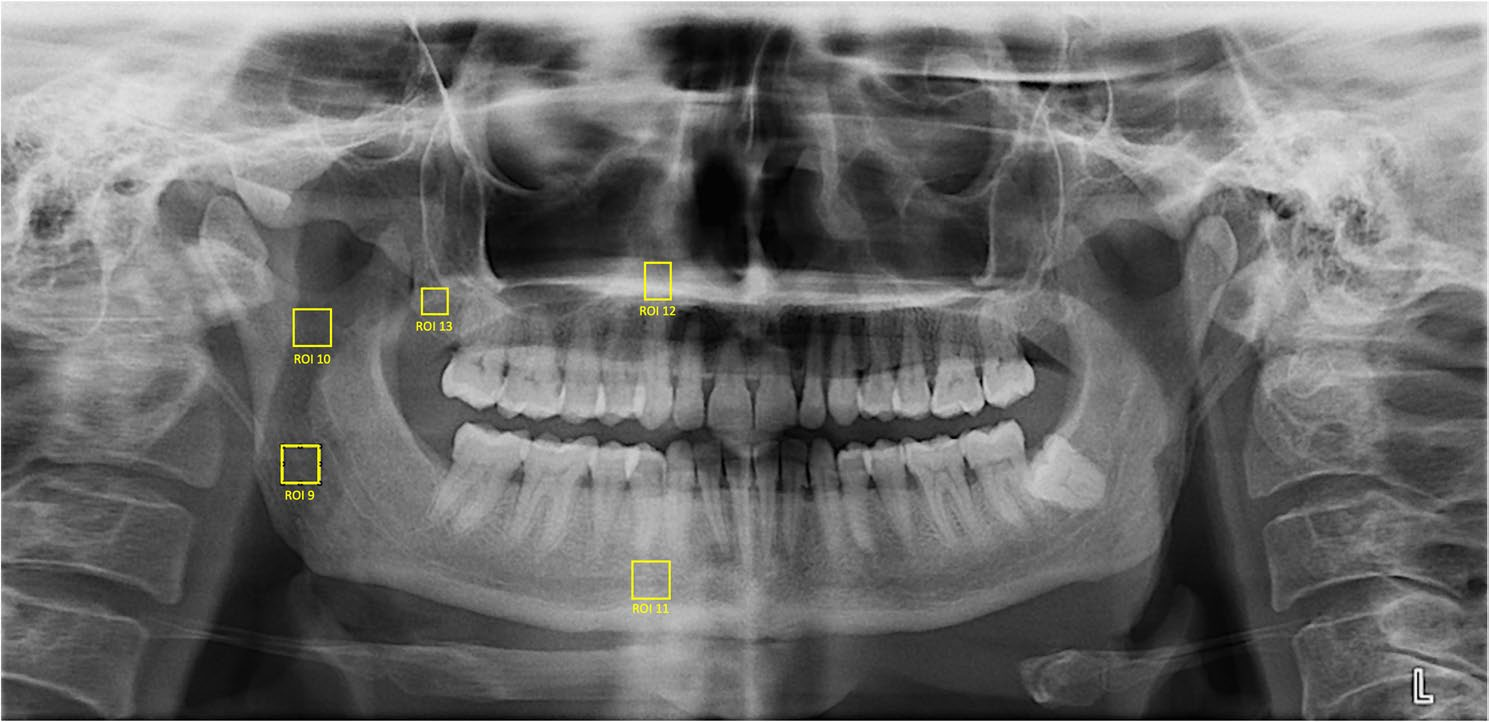
Regions of interest (ROIs) identified on the OPG images. ROI 9: Mandibular foramen inferior region (70×70 px). ROI 10: Mandibular foramen superior region (70×70 px). ROI 11: Mental foramen medial region (70×70 px). ROI 12: Maxillary canine periapex (25×50 px). ROI 13: Maxillary tuberosity (50×50 px). ROI 14: Necrotic bone area (50×50 px, MRONJ group only)

**Fig. 3:**
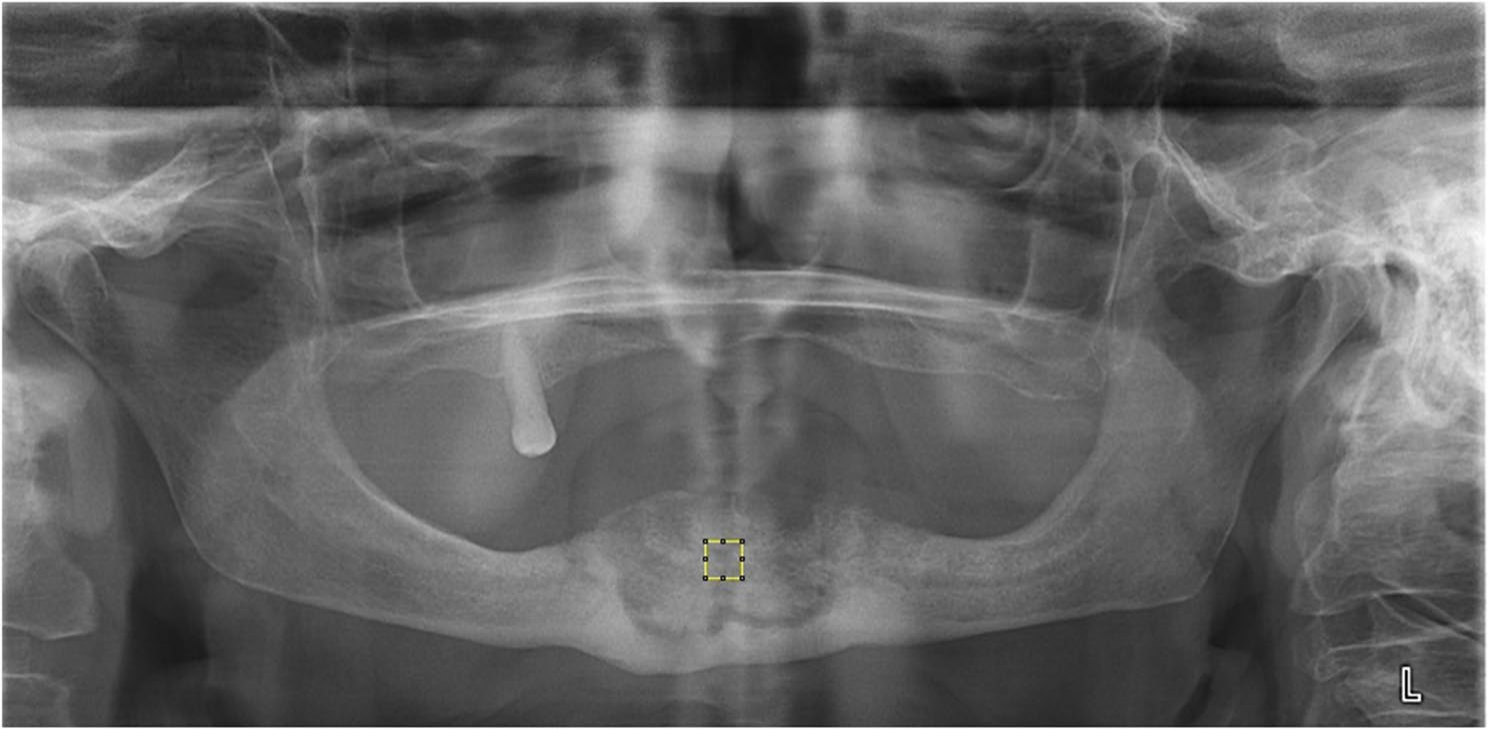
Selection of the ROI from the necrotic bone area on the OPG image (ROI 14)

For OPG images (Figs. 2 and 3):

- ROI 9 – Mandibular foramen inferior region (70 × 70 pixels).
- ROI 10 – Mandibular foramen superior region (70 × 70 pixels).
- ROI 11 – Mental foramen medial region (70 × 70 pixels).
- ROI 12 – Maxillary canine periapex (25 × 50 pixels).
- ROI 13 – Maxillary tuberosity (50 × 50 pixels).
- ROI 14 – Necrotic bone area (50 × 50 pixels, MRONJ group only).

Pixel sizes and ROI positions were standardized across all subjects to ensure reproducibility and to target trabecular bone exclusively, avoiding cortical interference. For the majority of OPG ROIs, the size was set to 70 × 70 pixels; however, in ROI 12, ROI 13, and ROI 14, anatomical limitations required smaller dimensions, which were adjusted to capture the maximum possible trabecular bone area without including cortical borders.

Fractal analysis was performed using ImageJ^®^ software (version 1.52p, NIH, Bethesda, MD, USA) and the box-counting method described by White and Rudolph [21]. In this method, the image is divided into squares of varying dimensions (2, 3, 4, 6, 8, 12, 16, 32, and 64 pixels). For each square size, the number of boxes containing trabeculae is calculated. These values are then plotted on a logarithmic scale, and a line of best fit is drawn. The slope of this line yields the FD value, reflecting the complexity of the trabecular structure [22].

**Fig. 4:**
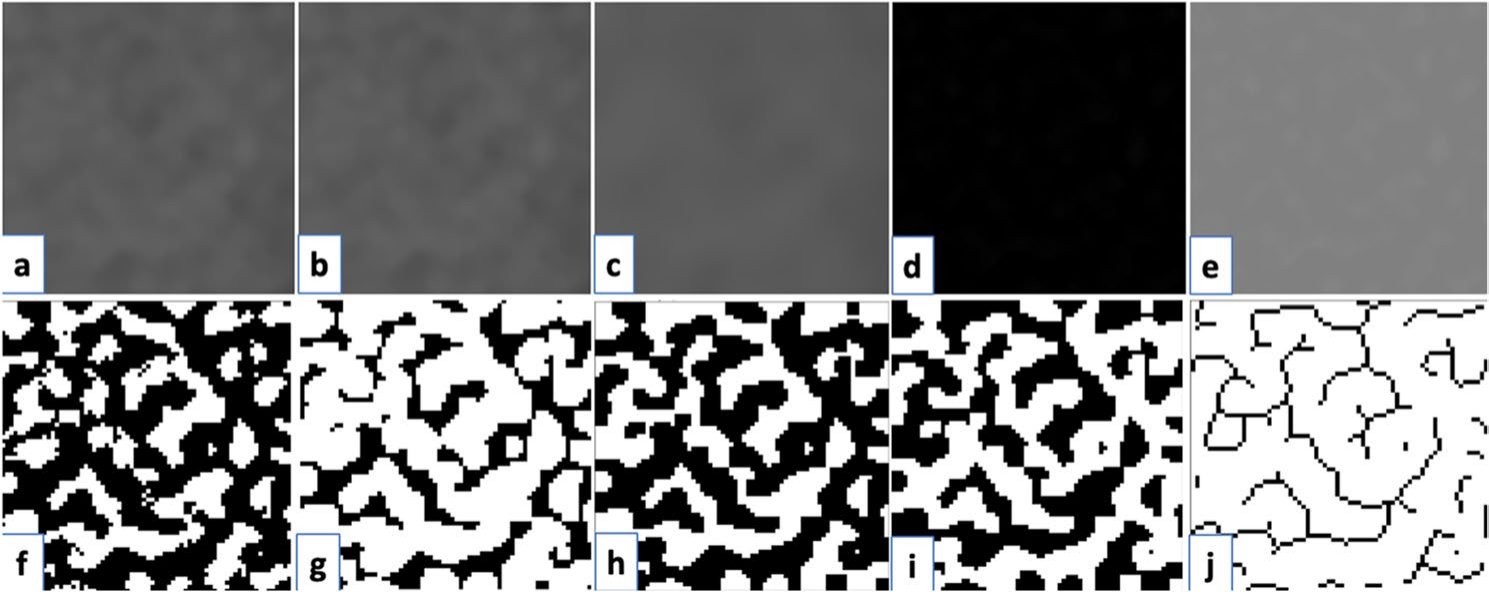
FB processing steps in OPG images. **a.** Cropping the determined ROI, **b.** Duplicating the cropped image, **c.** Blurring the duplicated image, **d.** Subtracting the blurred image from the cropped image, **e.** Adding a gray value of 128 to the resulting image, **f.** Binarizing the resulting image with a threshold value of 128, **g.** Applying an erosion process to the binarized image, **h.** Applying a dilation process, **i. **Applying an inversion process, **j.** Skeletonizing the inverted image

Each ROI was cropped to the predefined pixel dimensions and converted to 8-bit grayscale to enhance contrast. A Gaussian blur filter was then applied, and the blurred image was subtracted from the original, followed by adding a constant grayscale value of 128 to normalize intensity. The processed images were subsequently binarized to distinguish trabecular bone from the background and skeletonized to simplify the structure, enabling fractal dimension (FD) calculation (Fig. 4).

Statistical analyses were performed using IBM SPSS Statistics (version 20.0, IBM Corp., Armonk, NY, USA). Normality was assessed with the Shapiro–Wilk test. Group comparisons were made using independent t-tests or Mann–Whitney U tests, as appropriate. Pearson’s correlation coefficient evaluated associations between FD values and clinical variables (e.g., therapy duration). Categorical variables were compared using chi-square or Fisher’s exact tests. Continuous data are presented as mean ± standard deviation (SD) and categorical data as frequencies and percentages. Statistical significance was set at *p* < 0.05. Intraobserver reliability was assessed by repeating FD measurements for 11 randomly selected images after 15 days, with Cronbach’s alpha indicating strong agreement (α > 0.90).

## Results

A total of 54 participants were included: 26 MRONJ patients (mean age 66.34 ± 13.9 years; range 41–88) and 28 healthy controls (mean age 60.86 ± 7.7 years; range 41–76 years). The MRONJ group comprised 8 males and 18 females, whereas the control group included 13 males and 15 females. The mean duration of intravenous zoledronic acid therapy among MRONJ patients was 2.13 ± 0.95 years (Table 1). Comparisons of FD values between genders and correlations between age and FD values were conducted only in the control group, as trabecular bone alterations associated with MRONJ could confound these relationships.

**Table 1:** Demographic and clinical characteristics of the MRONJ and control groups

Characteristics	MRONJ (*n* = 26)	Control(*n* = 28)
Age (mean ± SD)	66,34 ± 13,9	60,86 ± 7,7
Age Range (years)	41–88	41–76
Gender (Male/Female)	8/18	13/15
Medication Use Duration (mean ± SD, years)	2,13 ± 0,95	-

*SD *standard deviation

### Comparison of FD values between genders

In the CBCT images of the control group, males had significantly higher FD values than females in three anatomical regions: superior to the mandibular foramen in the sagittal view (ROI 2, right; *p* = 0.005), superior to the inferior alveolar canal in the axial view (ROI 4, left; *p* = 0.012), and maxillary tuberosity in the coronal view (ROI 7, left; *p* = 0.025). No significant sex-related differences were observed in the remaining regions (*p* > 0.05). In the OPG images, no statistically significant gender differences in FD values were found in any anatomical region (*p* > 0.05; Table 2).

**Table 2:** FD values from CBCT and OPG images for the male and female participants in the control group

Imaging Method	ROI	Male Group (*n* = 13)	Female Group (*n* = 15)	*p*
CBCT	ROI 1- left	1.36 ± 0.03	1.36 ± 0.03	0.856
	ROI 1- right	1.37 ± 0.04	1.37 ± 0.02	0.995
	ROI 2- left	1.40 ± 0.03	1.39 ± 0.03	0.725
	ROI 2- right	1.39 ± 0.02	1.38 ± 0.03	0.005^*^
	ROI 3- left	1.38 ± 0.04	1.37 ± 0.03	0.847
	ROI 3- right	1.38 ± 0.03	1.37 ± 0.03	0.947
	ROI 4- left	1.38 ± 0.04	1.36 ± 0.02	**0.012** ^*^
	ROI 4- right	1.38 ± 0.02	1.36 ± 0.04	0.397
	ROI 5- left	1.36 ± 0.04	1.36 ± 0.03	0.610
	ROI 5- right	1.36 ± 0.01	1.36 ± 0.01	0.246
	ROI 6- left	1.38 ± 0.03	1.38 ± 0.04	0.375
	ROI 6- right	1.37 ± 0.03	1.38 ± 0.02	0.074
	ROI 7- left	1.39 ± 0.01	1.39 ± 0.03	0.025^*^
	ROI 7- right	1.37 ± 0.03	1.37 ± 0.03	0.969
OPG	ROI 9- left	1.33 ± 0.03	1.32 ± 0.03	0.751
	ROI 9- right	1.34 ± 0.02	1.31 ± 0.03	0.338
	ROI 10- left	1.34 ± 0.03	1.32 ± 0.02	0.107
	ROI 10- right	1.34 ± 0.04	1.32 ± 0.03	0.795
	ROI 11- left	1.33 ± 0.03	1.31 ± 0.03	0.980
	ROI 11- right	1.33 ± 0.04	1.30 ± 0.03	0.737
	ROI 12- left	1.35 ± 0.03	1.35 ± 0.03	0.976
	ROI 12- right	1.35 ± 0.03	1.36 ± 0.02	0.226
	ROI 13- left	1.48 ± 0.02	1.46 ± 0.03	0.242
	ROI 13- right	1.47 ± 0.02	1.47 ± 0.02	0.861

Datas are presented as the mean ± standard deviation

Group comparisons were performed using the independent t-test for normally distributed data and the Mann–Whitney U test for non-normally distributed data, as determined by the Shapiro–Wilk test

*FD* fractal dimension, *ROI* region of interest

^*^ Statistically significant at *p* < 0.05

### Comparison of FD values between the MRONJ and control groups

In the CBCT images, the control group exhibited significantly higher FD values than the MRONJ group in all regions, except for the medial to the mental foramen in the axial view on the left side (ROI 5-left, *p* = 0.116), where the difference was not statistically significant. In contrast, OPG images showed no statistically significant differences in FD values between the MRONJ and control groups in any region (*p* > 0.05; Table 3).

**Table 3:** Comparison of FD values between the MRONJ and control groups on CBCT and OPG images

Imaging Method	ROI	Control Group (*n* = 28)	MRONJ Group (*n* = 26)	*p*
CBCT	ROI 1- left	1.36 ± 0.02	1.34 ± 0.03	**0.018** ^*^
	ROI 1- right	1.37 ± 0.03	1.34 ± 0.04	**0.002** ^*^
	ROI 2- left	1.39 ± 0.03	1.37 ± 0.04	**0.005** ^*^
	ROI 2- right	1.39 ± 0.02	1.36 ± 0.03	**0.002** ^*^
	ROI 3- left	1.37 ± 0.03	1.34 ± 0.03	**0.001** ^*^
	ROI 3- right	1.38 ± 0.03	1.36 ± 0.03	**0.003** ^*^
	ROI 4- left	1.37 ± 0.03	1.33 ± 0.05	**0.003** ^*^
	ROI 4- right	1.37 ± 0.03	1.34 ± 0.04	**0.004** ^*^
	ROI 5- left	1.36 ± 0.04	1.34 ± 0.04	0.116
	ROI 5- right	1.36 ± 0.03	1.34 ± 0.03	**0.003** ^*^
	ROI 6- left	1.38 ± 0.03	1.35 ± 0.06	**0.031** ^*^
	ROI 6- right	1.37 ± 0.03	1.35 ± 0.04	**0.026** ^*^
	ROI 7- left	1.38 ± 0.02	1.34 ± 0.04	**0.000** ^*^
	ROI 7- right	1.37 ± 0.03	1.33 ± 0.04	**0.000** ^*^
OPG	ROI 9- left	1.33 ± 0.03	1.32 ± 0.03	0.176
	ROI 9- right	1.32 ± 0.03	1.32 ± 0.03	0.573
	ROI 10- left	1.33 ± 0.03	1.33 ± 0.03	0.620
	ROI 10- right	1.33 ± 0.03	1.33 ± 0.03	0.916
	ROI 11- left	1.32 ± 0.03	1.33 ± 0.04	0.981
	ROI 11- right	1.31 ± 0.04	1.32 ± 0.03	0.378
	ROI 12- left	1.35 ± 0.02	1.34 ± 0.04	0.212
	ROI 12- right	1.36 ± 0.02	1.35 ± 0.04	0.403
	ROI 13- left	1.47 ± 0.03	1.47 ± 0.05	0.843
	ROI 13- right	1.47 ± 0.02	1.47 ± 0.04	0.774

Datas are presented as mean ± standard deviation

Group comparisons were performed using the independent t-test for normally distributed data and the Mann–Whitney U test for non-normally distributed data, as determined by the Shapiro–Wilk test

*MRONJ* medication-related osteonecrosis of the jaw, *ROI* region of interest

^*^ Statistically significant at *p* < 0.05

### Correlation between drug use duration and FD values

All MRONJ patients had received intravenous zoledronic acid therapy, with a mean treatment duration of 2.13 ± 0.95 years. In CBCT images, FD values showed a significant negative correlation with drug usage duration in three regions: superior to the inferior alveolar canal in the axial view (ROI 4-right, *p* = 0.024), medial to the mental foramen in the axial section (ROI 5-left, *p* = 0.009), and the maxillary tuberosity in the coronal view (ROI 7-right, *p* = 0.024). No significant correlations were observed in the remaining ROIs (*p* > 0.05). In OPG images, none of the ROIs demonstrated a significant correlation between FD values and drug usage duration (*p* > 0.05; Table 4).

**Table 4:** Correlation between medication duration and FD values on CBCT and OPG images in the MRONJ group

Imaging Method	Group (*n* = 26)	Pearson Correlation Coefficient	*p*
CBCT	ROI 1- left	−0.068	0.741
	ROI 1- right	−0.140	0.495
	ROI 2- left	−0.012	0.953
	ROI 2- right	−0.016	0.937
	ROI 3- left	−0.143	0.485
	ROI 3- right	−0.297	0.141
	ROI 4- left	0.343	0.087
	ROI 4- right	−0.440	0.024*
	ROI 5- left	−0.499	0.009*
	ROI 5- right	−0.041	0.843
	ROI 6- left	0.084	0.682
	ROI 6- right	0.137	0.505
	ROI 7- left	0.116	0.573
	ROI 7- right	−0.442	0.024*
OPG	ROI 9- left	−0.134	0.513
	ROI 9- right	−0.097	0.639
	ROI 10- left	−0.144	0.482
	ROI 10- right	−0.357	0.073
	ROI 11- left	−0.276	0.173
	ROI 11- right	0.045	0.827
	ROI 12- left	0.035	0.865
	ROI 12- right	−0.056	0.787
	ROI 13- left	0.123	0.550
	ROI 13- right	−0.241	0.237

* Statistically significant at *p* < 0.05

### Comparison of FD values between exposed and nonexposed bone regions

In the MRONJ group, CBCT images revealed significantly lower FD values in the exposed necrotic bone region (ROI 8) compared to the other regions, except for the superior to the inferior alveolar canal in the axial plane (ROI 4, *p* > 0.05), where the difference was not statistically significant. In contrast, OPG images showed no statistically significant differences in FD values between the exposed necrotic bone region (ROI 14) and any other region (*p* > 0.05; Table 5).

**Table 5:** Comparison of FD values between ROI 8 on CBCT and ROI 14 on OPG and the non-necrotic ROIs in the MRONJ group

Imaging Method	ROI		*p*
CBCT (ROI 8 = 1.32 ± 0.02)	ROI 1- left	1.34 ± 0.04	0.013*
	ROI 1- right	1.34 ± 0.04	0.005*
	ROI 2- left	1.37 ± 0.04	0.000*
	ROI 2- right	1.36 ± 0.03	0.000*
	ROI 3- left	1.34 ± 0.03	0.001*
	ROI 3- right	1.36 ± 0.03	0.000*
	ROI 4- left	1.33 ± 0.05	0.102
	ROI 4- right	1.34 ± 0.04	0.022*
	ROI 5- left	1.34 ± 0.04	0.008*
	ROI 5- right	1.34 ± 0.03	0.016*
	ROI 6- left	1.35 ± 0.06	0.014*
	ROI 6- right	1.35 ± 0.04	0.001*
	ROI 7- left	1.34 ± 0.04	0.007*
	ROI 7- right	1.33 ± 0.04	0.035*
OPG (ROI 8 = 1.33 ± 0.04)	ROI 9- left	1.32 ± 0.03	0.449
	ROI 9- right	1.32 ± 0.03	0.526
	ROI 10- left	1.33 ± 0.03	0.292
	ROI 10- right	1.33 ± 0.03	0.678
	ROI 11- left	1.32 ± 0.03	0.316
	ROI 11- right	1.32 ± 0.03	0.635

Datas are presented as mean ± standard deviation

Group comparisons were performed using the independent t-test for normally distributed data and the Mann–Whitney U test for non-normally distributed data, as determined by the Shapiro–Wilk test

*ROI* region of interest, *CBCT *cone beam computed tomography, *OPG* orthopantomography

* Statistically significant at *p* < 0.05

** was made only with areas of the same size (70 × 70) as ROI 14 for the OPG images; therefore, ROI 12 (25 × 50) and ROI 13 (50 × 50) were not included in the comparison

## Discussion

MRONJ is a severe adverse effect linked to the use of antiresorptive and antiangiogenic agents, particularly bisphosphonates [4]. Although its occurrence is well-documented, early detection remains difficult because initial clinical manifestations and radiographic changes are often subtle and nonspecific [23].

This study aimed to evaluate trabecular bone alterations in MRONJ patients using fractal analysis of CBCT and OPG images, by comparing FD values between exposed necrotic and radiographically healthy bone regions, as well as with healthy controls. Unlike previous research that primarily described radiographic features such as osteosclerosis, osteolysis, and periosteal bone deposition in MRONJ [5, 17], our approach quantitatively assessed these changes through FD analysis. This method not only provided an objective measurement of trabecular complexity but also allowed the detection of subtle bone microarchitecture alterations that may precede the clinical manifestation of MRONJ. By directly contrasting FD values in different anatomical regions and imaging modalities, this study offers novel insights into the diagnostic potential of FD analysis for early detection and monitoring of MRONJ-related bone changes.

Fractal analysis is a mathematical method that quantifies structural complexity and has proven utility in evaluating bone microarchitecture [12, 21]. In this study, CBCT images revealed significantly lower FD values not only in necrotic areas but also in multiple regions of radiographically healthy-appearing bone in MRONJ patients compared to controls. This finding suggests that, beyond the macroscopic changes such as osteosclerosis and osteolysis described by Arce et al. [6], antiresorptive therapy may exert a systemic effect leading to subtle, widespread trabecular deterioration throughout the jawbone. This result is strongly consistent with the findings of Bachtler et al. [8], who also demonstrated significantly lower FD values in MRONJ patients, suggesting a loss of trabecular complexity. Conversely, our findings directly contrast with those of Torres et al. [7], who reported higher FD values in certain CBCT ROIs, attributing the increase to reactive bone formation around necrotic regions. We believe this disagreement highlights a critical methodological variability: differences in ROI selection, image processing parameters (voxel size), and imaging settings significantly influence the calculated FD value. Unlike conventional radiographic assessment, fractal analysis offers a sensitive, quantitative means of detecting these subclinical alterations, thereby extending the work of Torres et al. [7] and Bachtler et al. [8] by demonstrating that FD changes are not confined to clinically affected areas. In contrast, OPG-derived FD values showed only a downward trend without reaching statistical significance, consistent with the observations of Demiralp et al. [24] and Şahin et al. [25], who questioned the diagnostic reliability of OPG-based FD analysis for MRONJ. While previous studies have explored associations between FD and other bone quality indicators—such as bone mineral density (BMD), Hounsfield units (HU), and serum CTx levels [26, 27] - these relationships remain inconclusive. Taken together, our findings strengthen the case for CBCT-based FD analysis as a valuable quantitative adjunct to conventional imaging and biochemical evaluations, with the potential to detect early microarchitectural changes in MRONJ before overt clinical signs emerge.

Our results demonstrated that CBCT exhibited higher sensitivity than OPG in detecting trabecular alterations, as determined through FD analysis. This superiority is likely attributable to CBCT’s three-dimensional imaging capability and reduced superimposition artifacts, rather than being a generalizable advantage across all diagnostic applications. While these findings are in line with the conclusions of Saberi et al. [18] and Magat et al. [19], who highlighted the superiority of CBCT in visualizing trabecular patterns [28, 29], they should be interpreted with caution. Differences in statistical significance may also arise from methodological factors, including ROI selection, image acquisition parameters, and analysis protocols. In contrast, OPG remains a widely available, low-radiation, and cost-effective imaging modality, but its diagnostic accuracy in trabecular assessment is inherently limited by superimposition and distortion, as noted by Demiralp et al. [24] and Gümüşsoy et al. [30]. In clinical practice, OPG may still serve as a practical tool for initial screening, whereas CBCT can be reserved for cases requiring detailed trabecular evaluation.

Our findings on subclinical FD changes underscore the value of early and objective diagnostic tools in MRONJ, which is crucial given the complexity of current management strategies. Recent literature highlights a growing focus on adjunctive therapies aimed at improving healing outcomes following surgical debridement. These interventions include the use of autologous platelet concentrates (APCs), such as platelet-rich fibrin (PRF) and platelet-rich plasma (PRP), which are applied to enhance tissue regeneration and wound closure. Furthermore, emerging evidence supports the use of ozone infiltration combined with piezoelectric surgery, or conservative laser-assisted treatments (such as low-level laser therapy, LLLT), as safe and beneficial adjunctive options. Importantly, these therapeutic advancements are often studied in the context of various risk factors, such as the type of systemic drug administration, reinforcing the central importance of quantitative, non-invasive diagnostic monitoring—like CBCT-based FA—to accurately assess bone viability and guide surgical planning, thereby optimizing the success of these emerging treatments [31–34].

Interestingly, while Torres et al. [7] reported higher FD values in certain CBCT ROIs of MRONJ patients—attributed to reparative bone formation around necrotic regions—both our study and that of Bachtler et al. [8] consistently demonstrated lower FD values in MRONJ patients compared to controls, suggesting a loss of trabecular complexity due to necrosis. These discrepancies may be explained by differences in ROI selection, voxel size, and imaging settings, underscoring the importance of standardized protocols in future FD research. The reduced FD values observed in our MRONJ cohort likely reflect the disruption of trabecular microarchitecture caused by both osteonecrosis and antiresorptive therapy [35]. Similar reductions have been documented in other bone-degenerative conditions, such as sickle cell anemia and temporomandibular joint disorders, as reported by Demirbaş et al. [24] and Arsan et al. [36]. Together, these observations strengthen the evidence for FD analysis as a noninvasive and quantitative tool for assessing trabecular integrity in both systemic and localized bone pathologies, while emphasizing the need for further validation of its diagnostic reliability across diverse clinical contexts.

Our study also examined the relationship between zoledronate treatment duration and trabecular bone structure, as assessed by FD analysis. In CBCT images, a significant negative correlation was found in selected ROIs, indicating that longer bisphosphonate exposure may exacerbate trabecular deterioration. This aligns with the pathophysiological understanding that prolonged suppression of bone turnover can lead to compromised microarchitecture. Interestingly, Apolinario et al. [37] observed increased FD values in osteogenesis imperfecta patients after pamidronate therapy, suggesting that the effects of antiresorptive agents on trabecular patterns are not uniform and may depend on disease-specific remodeling dynamics and drug mechanisms [38]. Given that MRONJ evolves through distinct stages with differing patterns of trabecular change, FD values are likely to vary according to disease severity. This highlights a potential application of FD analysis not only for early detection but also for longitudinal monitoring of MRONJ progression. Future studies should therefore consider stratifying patients by MRONJ stage and treatment history, enabling a more precise evaluation of temporal changes in trabecular architecture and the prognostic utility of FD metrics.

Although sex was not a primary variable of interest in this study, evaluating its relationship with FD values provides important context for interpreting variability in trabecular bone assessments. Understanding whether FD metrics remain consistent across demographic subgroups is essential for determining their generalizability in clinical practice. Significant sex-related differences were observed in selected ROIs within the control group; however, these were not consistent across all anatomical sites, suggesting that the influence of sex on FD may be site-specific rather than systemic. These findings highlight the need for larger, stratified studies to better clarify the impact of demographic factors on FD values, particularly in the context of MRONJ.

FD analysis shows promise as a complementary tool to conventional imaging by providing objective, quantitative metrics for assessing trabecular alterations in MRONJ. The unique contribution of our study lies in its direct, head-to-head comparison of FA performance between CBCT and OPG in the same patient cohort, demonstrating CBCT’s superior sensitivity in revealing widespread microarchitectural changes in radiographically normal-appearing bone. Our findings indicate its potential not only for detecting established lesions but also for revealing microarchitectural changes in radiographically normal-appearing bone, suggesting a possible role in early detection and individualized treatment planning. However, several limitations should be considered.

FD analysis has several methodological constraints. CBCT-related limitations include the potential for artifacts from metallic restorations, which can compromise image quality for FD calculation, although exclusion criteria were applied to minimize this risk. Furthermore, the voxel size (400 μm), though high-resolution, remains lower than that of micro-CT and thus limits the capture of true microarchitectural detail. Methodological limitations specific to FA include the non-uniformity of ROI selection: while standardization was rigorously pursued for all CBCT images, anatomical necessity required variable ROI dimensions for specific OPG regions (ROI 12, 13, 14). This methodological variance, along with the inherent sensitivity of FD values to image acquisition parameters (e.g., exposure settings), potentially impacts the comparability of OPG results.

Nevertheless, FA’s immediate clinical translation faces several barriers. FD analysis is not yet a standardized, commercially available tool in dental imaging software. For clinical integration, widespread implementation will require international consensus on acquisition parameters (kVp, mA, voxel size), automated and validated ROI selection protocols, and robust thresholding techniques to reduce operator dependency and ensure reproducible decision-making.

The relatively modest sample size, although comparable to similar studies, may limit statistical power and generalizability. Baseline BMD was not assessed using dual-energy X-ray absorptiometry (DEXA), leaving natural age- and hormone-related variations—particularly in postmenopausal women—unaccounted for. The retrospective design also carries risks of selection bias, incomplete data capture, and limited control over confounding factors such as treatment adherence, comorbidities, and drug protocol variations. Furthermore, as all patients had stage 2 or 3 MRONJ, the lower FD values in radiographically healthy-appearing bone should be interpreted as reflecting systemic effects of antiresorptive therapy rather than definitive early predictors of lesion onset.

Prospective, longitudinal studies with larger and more diverse cohorts, standardized imaging protocols, quantitative bone assessments (e.g., DEXA), and inclusion of early-stage or high-risk patients are essential to validate the diagnostic potential of FD analysis and to optimize its integration into clinical workflows.

## Conclusions

CBCT-based fractal dimension analysis revealed significantly lower trabecular complexity in MRONJ patients compared with healthy controls, whereas OPG showed no significant differences. These findings highlight the superior sensitivity of CBCT for detecting microarchitectural changes and support the potential of FD analysis as a noninvasive, quantitative adjunct in MRONJ evaluation. However, methodological standardization and validation in larger, prospective cohorts are needed before its integration into routine clinical practice.

## Data Availability

The datasets generated and/or analysed during the current study are available from the corresponding author on reasonable request.

## Abbreviations

BMD: Bone mineral density
CBCT: Cone-beam computed tomography
CTx: C-terminal telopeptide of type ı collagen (serum marker of bone resorption)
DEXA: Dual-energy x-ray absorptiometry
FD: Fractal dimension
FA: Fractal analysis
FOV: Field of view
HU: Hounsfield unit
MRONJ: Medication-related osteonecrosis of the jaw
OPG: Orthopantomogram / panoramic radiograph
ROI: Region of interest
SD: Standard deviation
SPSS: Statistical package for the social sciences
